# A Scoping Review of the Barriers and Facilitators to Implementing Small‐Scale Residential Dementia Care

**DOI:** 10.1002/alz.086308

**Published:** 2025-01-09

**Authors:** Robyn A Lewis, Diane Gibson, Stephen Isbel, Kasia Bail, Nathan M D'Cunha, Centre for Ageing Research and Translation, University of Canberra, Bruce, ACT, 2617

**Affiliations:** ^1^ University Of Canberra, Canberra, ACT Australia; ^2^ University of Canberra, Bruce, ACT Australia; ^3^ Faculty of Health, University of Canberra, Bruce, ACT Australia

## Abstract

**Background:**

There is a need to understand the benefits and limitations of innovative models of dementia care to ensure models meet the needs of people living with dementia, their families and staff. The aim of this scoping review was to explore and synthesise the barriers and facilitators to the widespread implementation of small‐scale residential dementia care.

**Method:**

A scoping review was conducted in 2023 in MEDLINE, CINAHL, PsycINFO, Scopus, Web of Science, and CENTRAL to identify empirical, peer‐reviewed studies, published in English from database inception to October 2023. Studies of small‐scale residential dementia care with 6‐15 people were included.

**Result:**

Forty‐four studies were identified and synthesised into five focus areas for investigation: People with dementia (n = 19), families (n = 2), staff (n = 12), the built environment (n = 8) and other studies with a focus on a program of care or studies with two or more study populations (n = 3). Studies were published between 1990 and 2023 and included quantitative (n = 29), qualitative (n = 13) and mixed methods (n = 2) approaches. Multiple barriers and facilitators were identified in each focus area that either support or limit the widespread implementation of small‐scale residential dementia care. A key facilitator is the presence of a clear philosophy of small‐scale care that guides the planning of a small‐scale setting or adaptation of a traditional care setting to incorporate principles of small‐scale care. The philosophy should be articulated through policies, communicated to stakeholder groups and implemented consistently in the delivery of care. A key barrier relates to the ability of the care organisation to adjust staff roles to deliver small‐scale care. With broader caring roles and greater decision‐making authority, organisational changes are required to staff ratios, training and education to empower staff to deliver this care.

**Conclusion:**

Small‐scale residential dementia care has the potential to benefit people with dementia, their families, the staff and the wider community. Through the establishment of a clear philosophy of small‐scale care, there is an opportunity to place the person with dementia at the centre of care, provide access to meaningful and appropriate activities of daily living, create a home‐like environment and prioritise relationship building at all levels.

